# Prevalence and Factors Associated With Poor Glycaemic Control in Type 2 Diabetes Mellitus Patients: A Single‐Centre, Hospital‐Based Cross‐Sectional Study in Jhenaidah District, Bangladesh

**DOI:** 10.1002/edm2.70312

**Published:** 2026-08-19

**Authors:** Md. Sakhawot Hossain, Rakibul Islam, Md. Moshiuzzaman Adnan, Md. Shahadoth Hossain, Quazi Mumtarina Tabassum, Md. Hasan Al Banna, Md. Shovon Al‐Fuad, Tasniya Akter, Muhammad Tayyab Arshad, Md. Amanullah, Fardib Mahbub, Tanvir Ahmad

**Affiliations:** ^1^ Department of Nutrition and Food Engineering Daffodil International University Dhaka Bangladesh; ^2^ Department of Nutrition and Food Technology Jashore University of Science and Technology Jashore Bangladesh; ^3^ Faculty of Nutrition and Food Science Patuakhali Science and Technology University Patuakhali Bangladesh; ^4^ Functional Food and Nutrition Program, Faculty of Agro‐Industry Prince of Songkla University Hatyai Songkhla Thailand

**Keywords:** Bangladesh, diabetes complications, diabetes mellitus, glycaemic control, lifestyle modification

## Abstract

**Background:**

Poor glycaemic control is a significant public health concern among patients with Type 2 diabetes mellitus (T2DM), contributing to the progression of diabetes‐related complications. This study aimed to assess the prevalence of and factors associated with poor glycaemic control among patients with T2DM attending a single specialised diabetic hospital in Jhenaidah, Bangladesh.

**Methods:**

A hospital‐based cross‐sectional study was conducted among 497 patients with T2DM attending the outpatient department of Jhenaidah Diabetic Hospital, Bangladesh, from October 2023 to April 2024. Eligibility required a recent glycated haemoglobin (HbA1c) result within the preceding 3 months. Data were collected through face‐to‐face interviews, review of medical records, and anthropometric measurements. Modified Poisson regression with robust variance was used to identify factors associated with poor glycaemic control; adjusted prevalence ratios (aPRs) with 95% confidence intervals (CIs) were estimated, and statistical significance was set at *p* < 0.05.

**Results:**

The prevalence of poor glycaemic control among patients with T2DM was 79.1% (95% CI, 75.3%–82.4%). Overall, 72.4% of participants had at least 1 comorbidity, most often overweight/obesity (63.4%) (composite measure: overweight/obesity, hypertension, and reduced eGFR), and 81.1% had at least 1 diabetes‐related complication, most commonly neuropathy (59.6%). Patients with a diabetes duration of 6–10 years had a higher prevalence of poor control (aPR = 1.13, 95% CI, 1.01–1.27, *p* = 0.037). Those with 1 comorbidity (aPR = 1.20, 95% CI, 1.05–1.36, *p* = 0.007) and 2 or more comorbidities (aPR = 1.25, 95% CI, 1.09–1.43, *p* = 0.002) also had a higher prevalence of poor glycaemic control.

**Conclusion:**

Longer diabetes duration and comorbidity burden were significantly associated with poor glycaemic control. Closer monitoring of these patients may improve outcomes, but larger multicentre studies are needed to confirm the findings. As a single‐centre, cross‐sectional study, the estimate is clinic‐based rather than population‐representative, and the associations cannot be interpreted as causal.

## Graphical abstract

Poor glycaemic control was highly prevalent among Bangladeshi patients with Type 2 diabetes mellitus. Longer diabetes duration and increased comorbidity burden were significant predictors of poor glycaemic control, highlighting the urgent need for targeted lifestyle interventions, regular monitoring, and improved health awareness to reduce diabetes‐related complications and improve patient outcomes.

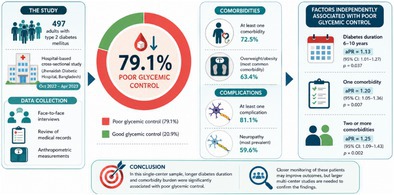

## 1 Introduction

Type 2 diabetes mellitus (T2DM) is a chronic, progressive metabolic disorder characterized by persistent hyperglycaemia resulting from a combination of insulin resistance and progressive pancreatic β‐cell dysfunction, leading to inadequate insulin secretion relative to the body's metabolic demands [1, 2]. Over the past few decades, T2DM has become a leading cause of morbidity and mortality worldwide, with its prevalence increasing rapidly due to urbanisation, lifestyle changes, and an ageing population [2, 3]. In Bangladesh, the burden of T2DM is particularly concerning, as it affects a substantial portion of the adult population and contributes significantly to the national healthcare burden [2]. Poor glycaemic control in patients with T2DM is a major factor contributing to the development of associated complications, which include cardiovascular diseases, diabetic retinopathy, neuropathy, and chronic kidney disease (CKD) [4]. Glycaemic control is typically measured by HbA1c, which provides a three‐month average of blood glucose levels [5]. The American Diabetes Association (ADA) recommends maintaining an HbA1c level below 7% for optimal diabetes control [6]. However, maintaining good glycaemic control remains a challenge for many patients with T2DM, especially in low‐ and middle‐income countries (LMICs) like Bangladesh, where healthcare access, diabetes awareness, and effective treatment options may be limited [7]. Globally, the prevalence of poor glycaemic control among patients with T2DM is alarmingly high. Multiple studies across different regions provide robust evidence for this claim. Prevalence rates of poor glycaemic control range from 45.2% to 93% [8]. Specific regional studies highlight the widespread nature of this issue: 74.9% in Saudi Arabia [9], 68.3% in Ethiopia [10], and 59.2% in Malaysia [11]. Even more concerning, a systematic review of insulin‐treated patients found that only 26.02% achieved good glycaemic control [12]. In Bangladesh, the situation is equally alarming. A cross‐sectional study by Reza et al. found that approximately 87.6% of patients with T2DM in the country had poor glycaemic control, based on data from 732 participants [13]. This high prevalence has been attributed to several factors commonly observed in Bangladesh, including poor medication adherence, inadequate diabetes‐related knowledge, limited access to specialized diabetes care, low socioeconomic status, unhealthy dietary practices, and insufficient physical activity, all of which compromise effective glycaemic management [14, 15, 16]. Several factors contribute to poor glycaemic control in patients with T2DM, which can be broadly categorized into sociodemographic, clinical, and lifestyle‐related variables. Age, gender, education level, and socioeconomic status have been shown to significantly affect the management of T2DM. Older adults are at greater risk of poor glycaemic control due to the natural decline in insulin sensitivity and the higher likelihood of developing comorbidities [17]. Similarly, low educational attainment and low socioeconomic status have been identified as barriers to effective diabetes management, as they are often associated with limited access to healthcare, lower health literacy, and poor adherence to lifestyle modifications and medication regimens [18]. The duration of diabetes is another critical factor influencing glycaemic control. Longer duration of diabetes is often associated with progressive deterioration of pancreatic β‐cell function, making it harder to maintain tight glycaemic control [19].

Furthermore, the presence of comorbidities such as hypertension, dyslipidemia, and chronic kidney disease significantly complicates diabetes management. Patients with comorbidities often have a higher medication burden, which can lead to poor adherence and suboptimal glycaemic control [20]. Lifestyle factors, including physical activity, diet, and smoking, also play a significant role in glycaemic control. Regular physical exercise is associated with improved insulin sensitivity and better blood sugar regulation [20]. However, many patients with T2DM, particularly in rural Bangladesh, are sedentary due to factors such as lack of access to safe recreational spaces, limited time, and cultural norms [21]. Similarly, dietary habits, such as high carbohydrate intake, excessive consumption of refined sugars, and low fibre intake, contribute to poor glycaemic control [22]. Smoking, which is prevalent among a significant portion of the Bangladeshi population, also worsens glycaemic control by increasing insulin resistance and impairing vascular health, further complicating diabetes management [14].

Given the high prevalence of T2DM and the significant burden of poor glycaemic control in Bangladesh, this study aims to assess the prevalence of poor glycaemic control among patients with T2DM in Jhenaidah, a district that may be underrepresented in current diabetes research. Additionally, the study seeks to identify sociodemographic, clinical, and lifestyle factors associated with poor glycaemic control in this population. The findings from this study are expected to contribute to a deeper understanding of the factors associated with poor glycaemic control among patients attending a specialised diabetic hospital in this setting and may help inform locally relevant clinical care, while recognising that a single‐centre, clinic‐based sample cannot be assumed to represent district‐level, rural, or national populations. By focusing on factors such as diabetes duration, comorbidities, education level, and lifestyle choices, this study will provide insights into the multifactorial nature of poor glycaemic control and highlight areas for intervention.

## 2 Methods

### 2.1 Study Design

A hospital‐based cross‐sectional study was conducted among patients diagnosed with T2DM attending the outpatient department of Jhenaidah Diabetic Hospital, Jhenaidah, Bangladesh. The study was carried out over a period of approximately six months, from 5 October 2023 to 1 April 2024. The study was designed, conducted, and reported in accordance with the Strengthening the Reporting of Observational Studies in Epidemiology (STROBE) guidelines for cross‐sectional studies.

### 2.2 Study Setting

**FIGURE 1:**
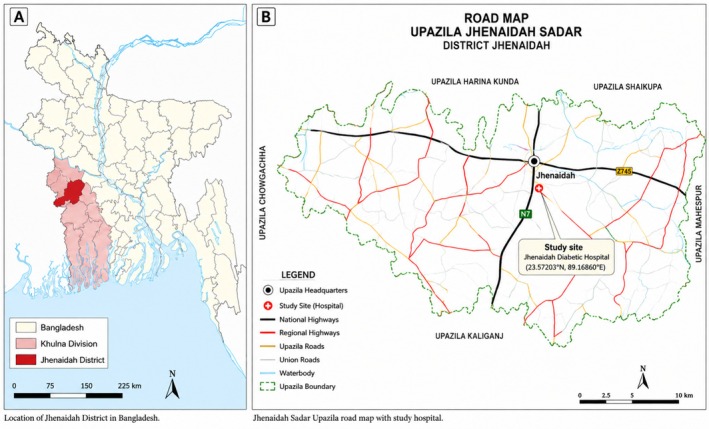
Geographic location of the study area.

This study was conducted at Jhenaidah Diabetic Hospital, a privately owned, specialised diabetes medical facility registered under the Facility Registry of the Ministry of Health and Family Welfare, Government of Bangladesh. The hospital is affiliated with the local diabetic association and operates as a dedicated referral centre for diabetes management in the region. It is situated in Jhenaidah Sadar Upazila, within the Jhenaidah district of Bangladesh, approximately 196 km southwest of the capital city, Dhaka (Figure 1). Jhenaidah Sadar Upazila covers a total area of 470.11 km^2^ and, according to the 2022 Bangladeshi census, has a population of 543,906 with a literacy rate of 75.18% among individuals aged 7 and above [23]. The hospital provides a comprehensive range of diabetes‐related clinical services, including outpatient consultations, diagnostic investigations, and disease management support for patients with diabetes and associated complications. Patients diagnosed with diabetes are frequently referred to this facility by local hospitals and community clinics, making it a well‐established point of care for a geographically and clinically diverse patient population.

The selection of this hospital as the study site was guided by several considerations. As a specialised diabetes facility with a consistently high volume of treatment‐seeking patients, it offered an efficient and practical setting for recruiting participants meeting the study's eligibility criteria. Furthermore, its status as a referral centre provided access to patients with varying degrees of diabetes severity, duration, and complication profiles. Prior to data collection, formal written permission was obtained from the hospital authority. The data collection and recruitment procedures were conducted independently of hospital staff, relying entirely on the voluntary participation and informed consent of individuals present at the facility for their routine outpatient care.

### 2.3 Sample Size Determination

The sample size for this study was determined using Cochran's single population proportion formula, *n* = $\frac{Z^{2}\times p\left(1-p\right)}{d^{2}}$, where *Z* = 1.96 for a 95% confidence interval (CI), a margin of error *d* = 0.05, and *p* represents the prevalence of inadequate glycaemic control (71.8%) among patients with Type 2 diabetes mellitus, as reported in a prior study conducted in Bangladesh [15]. For transparency, the base sample was calculated as *n* = (1.96)^2^ × 0.718 × (1–0.718)/(0.05)^2^ ≈311; after adjustment for 10% non‐response (311/0.9), this yielded the minimum required sample of approximately 346. A total of 497 participants were included in the final analysis, exceeding the minimum required sample size and thereby improving the precision of the prevalence estimate.

### 2.4 Participant Recruitment

**FIGURE 2:**
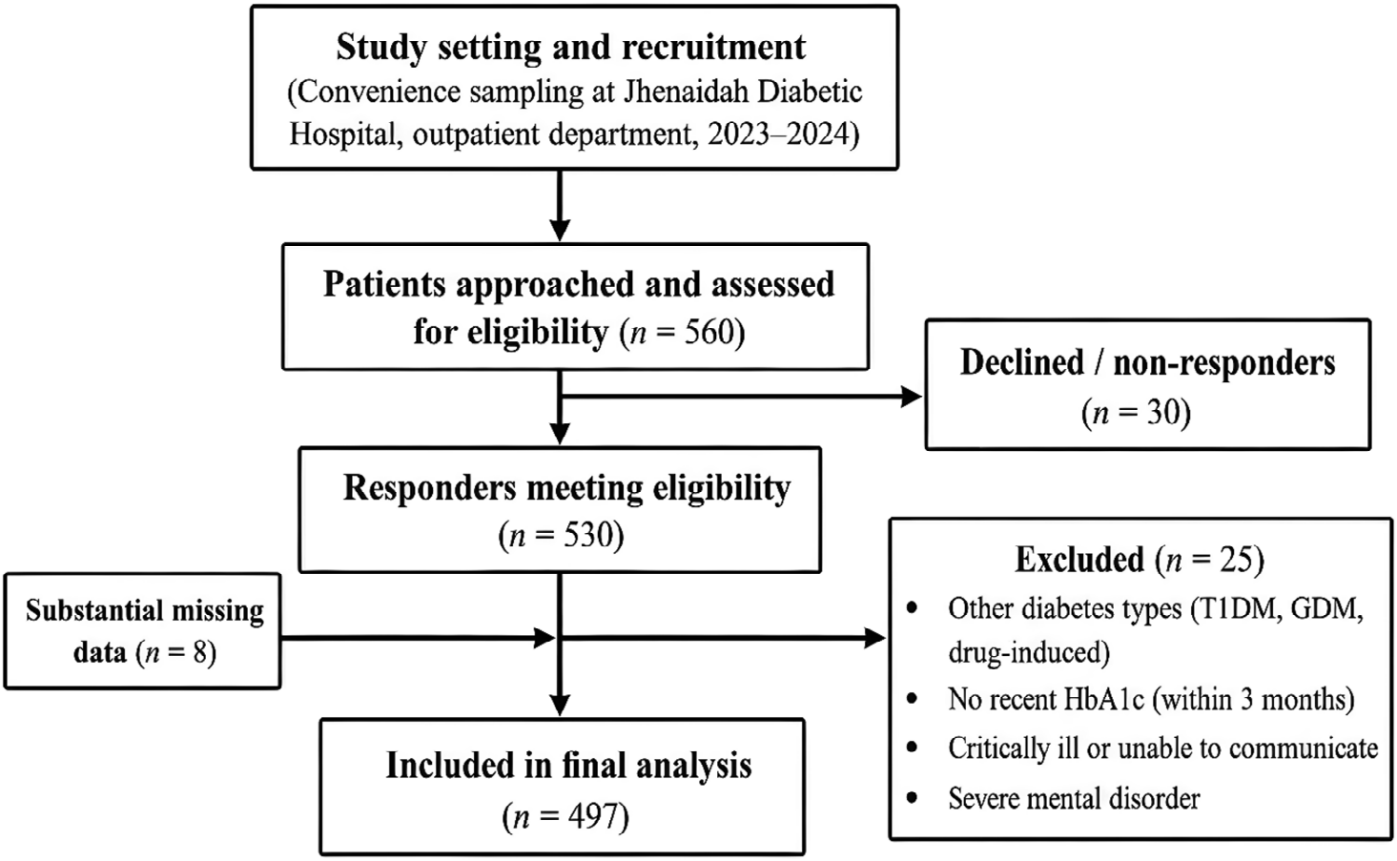
Flow diagram of participant recruitment and selection.

Eligible participants were recruited consecutively using a convenience sampling technique from the outpatient department (OPD) of Jhenaidah Diabetic Hospital during the study period. Patients who met the eligibility criteria and provided written informed consent were enrolled until the required sample size was achieved. The principal investigator and trained research assistants approached potential participants in the waiting area after they had completed their consultation with the attending physician. The study objectives and voluntary nature of participation were explained, and those who agreed to participate were screened for eligibility before being interviewed. Recruitment was carried out on regular clinic days throughout the study period, during outpatient working hours, whenever the research team was present. The flow of participants through recruitment, eligibility assessment, and inclusion is shown in Figure 2.

#### 2.4.1 Inclusion Criteria

To be eligible for participation, patients were required to meet all of the following criteria:

- Male or female patients aged 18 years or older.
- Registered as an outpatient with a confirmed diagnosis of T2DM for a minimum duration of 1 year
- Possession of recent glycated haemoglobin (HbA1c) laboratory test results, obtained no more than 3 months prior to the interview date
- Willingness to provide informed consent and participate in the study

#### 2.4.2 Exclusion Criteria

Patients were excluded from the study if they met any of the following conditions:

- Diagnosed with other forms of diabetes, including Type 1 diabetes mellitus, gestational diabetes, or drug‐induced diabetes mellitus
- Critically ill or medically unstable at the time of recruitment
- Presenting with psychotic conditions or severe mental health disorders
- Unable to communicate verbally and complete the interview process

### 2.5 Variables and Measurements

Data were collected through three complementary approaches: face‐to‐face interviews using a structured questionnaire, review of available medical records, and clinical assessments. Sociodemographic and lifestyle‐related variables were collected directly from participants through face‐to‐face interviews. Clinical and biochemical variables, including HbA1c values, were obtained through review of participants' medical records and laboratory reports. In instances where medical records did not contain information on a particular variable, the data were recorded as missing and excluded from the respective analysis, as detailed in the statistical methods section.

Three well‐trained research assistants were involved in the data collection process. Before implementing the data collection procedure, they were trained by the principal investigator (PI) on the study design and objectives, interview techniques, anthropometric measurements, review of medical records, and research ethics. The structured questionnaire was developed following a comprehensive review of relevant published literature on glycaemic control and associated factors among patients with T2DM, and captured information on sociodemographic characteristics, lifestyle behaviours, clinical history, and diabetes management practices [14].

#### 2.5.1 Glycaemic Control

The glycaemic control status of patients with T2DM was the outcome measure of the present study, assessed using HbA1c. With participant approval, the most recent HbA1c values (from within the past 3 months) were extracted from clinical laboratory reports contained in the participants' medical records. Glycaemic control was defined as good if HbA1c was below 7% and poor if HbA1c was ≥ 7%, according to the American Diabetes Association (ADA) criteria [6].

#### 2.5.2 Comorbidities and Complications

Data on comorbidities were obtained from the individuals' medical records. Hypertension (HTN) was characterized by a systolic blood pressure of ≥ 140 mmHg or a diastolic blood pressure of ≥ 90 mmHg, and/or an ongoing use of antihypertensive medications [6]. Reduced eGFR was identified from patients' medical records. Reduced eGFR was defined as an estimated glomerular filtration rate (eGFR) < 60 mL/min/1.73 m^2^, calculated using the 2021 CKD‐EPI equation, consistent with established clinical guidelines [24]. Because eGFR was based on laboratory values recorded at a single time point and persistence over at least 3 months could not be verified, this variable is more accurately interpreted as reduced eGFR rather than confirmed chronic kidney disease. Body weight and height were measured to the nearest 0.1 kg and 0.1 cm, respectively, employing established methods [25]. BMI was measured as weight (kg)/height (m^2^). Asian‐specific BMI cutoffs were used to define overweight/obesity (BMI ≥ 23.0 kg/m^2^) [26]. For the multivariable analysis, a composite comorbidity count was derived by summing the presence of hypertension, reduced eGFR, and overweight/obesity, and was categorized as no comorbidity, one comorbidity, or two or more comorbidities. Because these conditions differ in clinical significance, the composite count was used only to summarize overall comorbidity burden, and the individual‐component results (Table 5) should be regarded as the primary comorbidity analysis. Information on diabetes‐related complications, including diabetic retinopathy, diabetic neuropathy, foot ulcer, and stroke, was extracted from patients' medical record books based on documented physician diagnoses [27]. Only complications previously diagnosed and documented by the treating physicians or relevant specialists were considered in the analysis.

#### 2.5.3 Sociodemographic and Other Variables

The questionnaire comprised several domains, including sociodemographic, clinical, and lifestyle‐related variables. Sociodemographic characteristics included age, gender, education, residence, religion, occupation, and monthly family income. Clinical characteristics encompassed duration and family history of diabetes. Lifestyle‐related variables included sleep duration, smoking, and exercise. To measure physical exercise (PE) among patients with T2DM, a single item was applied to assess PE: ‘Do you regularly participate in physical exercise?’ The possible answers were 0 = ‘no’ or 1 = ‘yes.’ Based on this question, we dichotomized PE into two categories: irregular and regular. Because physical exercise was captured with a single yes/no item, this variable reflects only self‐reported regular versus irregular exercise and does not establish weekly duration, frequency, or intensity; it therefore cannot determine whether participants met the American Diabetes Association recommendation of at least 150 min of moderate‐intensity activity per week over at least 3 days for adults with T2DM [28].

### 2.6 Questionnaire Development and Pre‐Testing

A structured questionnaire was developed based on a review of relevant literature and previously validated instruments, and was adapted to the objectives of the present study. Before the main survey, the questionnaire was pre‐tested among 40 patients with T2DM attending a separate diabetic hospital that was not included in the main study. The pre‐test was conducted to evaluate the clarity, comprehensibility, acceptability, and administration time of the questionnaire. Based on participant feedback and discussion among the research team, minor revisions were made to improve the wording, clarity, and logical flow of several questions. The findings of the pre‐test and the corresponding revisions are presented in Table 1.

**TABLE 1:** Summary of the questionnaire pre‐test and revisions made to the final questionnaire.

Parameters	Pre‐test results	Revised in final questionnaire
Clarity and comprehension	Some participants found several questions unclear, particularly those containing medical terminology.	Questions were rephrased using simpler and more locally understandable language.
Acceptability	Participants did not express objections to questions related to disease history, smoking habits, or other personal health information. Some suggestions were provided regarding wording and flow.	No major changes were required. Minor wording adjustments were made after discussion within the research team.
Time required	The average time to complete the questionnaire during the pre‐test was approximately 20 min.	Interviewers were further trained to administer the questionnaire more efficiently, reducing the average completion time to approximately 15 min in the final survey.

### 2.7 Statistical Analysis

The data were manually scrutinized for errors and missing values by the data collector and subsequently re‐examined by the principal investigator (PI). Participant flow is summarized in Figure 2. Of the 560 patients approached, 497 met all eligibility criteria, provided informed consent, and had complete data on every variable entered in the regression model; these participants constituted the final analytic sample. A complete‐case analysis was therefore performed and no imputation was required.

All statistical analyses were performed using the Statistical Package for the Social Sciences (SPSS) version 25 (IBM Corp., Armonk, NY, USA) and R programming language (version 4.3.0). Descriptive statistics, including frequencies and percentages, were calculated for categorical sociodemographic, clinical, and lifestyle‐related variables, as well as for comorbidities and diabetes‐related complications. Inferential analysis was then conducted to identify factors associated with poor glycaemic control.

Because poor glycaemic control was a common outcome in this sample (prevalence of 79.1%), modified Poisson regression with robust (sandwich) variance estimators was used as the primary analytical approach to estimate crude and adjusted prevalence ratios (PRs) with 95% confidence intervals [29, 30]. When an outcome is highly prevalent, the odds ratio obtained from logistic regression diverges from, and overstates the magnitude of, the prevalence ratio; the modified Poisson approach therefore yields a more interpretable and less biased estimate of association for common binary outcomes [31]. Poor glycaemic control (HbA1c ≥ 7%) was coded as the outcome, with good control (HbA1c < 7%) as the reference; the reference categories for all categorical predictors are indicated in Table 4. Covariates were entered simultaneously in a single, prespecified model informed by prior literature and clinical reasoning rather than by automated stepwise selection or univariable *p*‐value screening. No interaction terms were included, and multicollinearity was assessed using variance inflation factors, with all values below the conventional threshold of 5, indicating no substantial collinearity. As a sensitivity analysis, a multivariable logistic regression model was fitted using the same set of covariates. Age and diabetes duration were modelled as categorical variables using the groupings shown in Table 4. Education was retained as the socioeconomic indicator; occupation and monthly income were not entered separately because they overlapped substantially with education and with each other, which would have introduced collinearity without adding independent information. Because diabetes‐related complications may lie on the causal pathway between prolonged hyperglycaemia and poor glycaemic control, their inclusion in the same model may introduce over‐adjustment, which is acknowledged as a limitation. The model demonstrated adequate fit based on the Omnibus test of model coefficients (*χ* ^2^ = 41.188, df = 20, *p* = 0.004) and the Hosmer–Lemeshow test (*χ* ^2^ = 12.718, df = 8, *p* = 0.122). The direction and statistical significance of the associations were consistent across the modified Poisson and logistic regression models, with differences observed only in the magnitude of the effect estimates; the overall study conclusions therefore remained unchanged irrespective of the regression model applied. A *p*‐value of less than 0.05 was considered statistically significant.

### 2.8 Ethical Considerations

Ethical approval for the study was obtained from the Ethical Review Committee of the Faculty of Biological Science and Technology, Jashore University of Science and Technology, on 25 September 2023 (ERC/FBST/JUST/2023–144), prior to the commencement of participant recruitment. The study was conducted in accordance with the principles of the Declaration of Helsinki. No blood samples were collected specifically for this research. Written informed consent was obtained from every participant before data collection; for participants who were unable to read or write, the study information and consent form were read aloud and consent was documented in accordance with the approved protocol. Participant confidentiality was strictly maintained, and all data were used only for research purposes.

## 3 Results

### 3.1 Sociodemographic and Lifestyle Characteristics of Participants

Among the 497 participants, slightly over half (54.9%) were male, and the largest age group was aged 50–59 years (32.4%). Nearly one‐quarter of the respondents (25.8%) were unable to read and write, and over half (52.3%) resided in rural areas. Approximately 39% of the participants were housewives, and 37.2% reported a monthly family income between Bangladeshi Taka (BDT) 10,000 and 20,000. Regarding clinical characteristics, nearly half of the patients (45.7%) had T2DM for 1–5 years, followed by 6–10 years (36.8%) and more than 10 years (17.5%). Additionally, 25.4% of participants reported having a family history of diabetes, and 54.7% were classified as overweight. Furthermore, 57.3% of participants reported a sleep duration of 6–8 h per day, 75.5% were non‐smokers, and 63.8% were involved in regular physical exercise (Table 2).

**TABLE 2:** Sociodemographic and lifestyle characteristics of the study participants (*N* = 497).

Characteristic	Frequency	%
Gender		
Male	273	54.9
Female	224	45.1
Age		
Below 40	59	11.9
40–49	128	25.8
50–59	161	32.4
60 and above	149	30.0
Education		
Unable to read and write	128	25.8
Primary	103	20.7
Secondary	138	27.8
Higher secondary	49	9.9
Graduation and above	79	15.9
Residence		
Urban	237	47.7
Rural	260	52.3
Religion		
Islam	419	84.3
Hindu	76	15.3
Christian	2	0.4
Occupation		
Day labour	32	6.4
Farmer	71	14.3
Service holder	90	18.1
Businessmen	83	16.7
Housewife	195	39.2
Retired/unemployed	26	5.2
Monthly family income (BDT)		
< 10,000	132	26.6
10,000–20,000	185	37.2
20,001–40,000	168	33.8
> 40,000	12	2.4
Duration of diabetes (years)		
1–5	227	45.7
6–10	183	36.8
> 10	87	17.5
Family history of diabetes		
No	371	74.6
Yes	126	25.4
BMI		
Underweight	18	3.6
Normal weight	164	33.0
Overweight	272	54.7
Obese	43	8.7
Sleep duration (hour)		
6–8	285	57.3
< 6	171	34.4
> 8	41	8.2
Smoking status		
Never	375	75.5
Current smoker	122	24.5
Physical exercise		
Regular	317	63.8
Irregular	180	36.2

*Note:* Values are presented as frequency (*n*) and percentage (%). Percentages are based on *N* = 497 unless otherwise indicated.

Abbreviations: BDT, Bangladeshi taka; BMI, body mass index; T2DM, Type 2 diabetes mellitus.

### 3.2 Prevalence and Factors Associated With Poor Glycaemic Control

The prevalence of poor glycaemic control among the T2DM patients was 79.1% (95% CI: 75.3%–82.4%). In the modified Poisson regression model with robust variance, a longer duration of diabetes and a higher comorbidity burden were significantly associated with poor glycemic control. Compared with patients diagnosed for 1–5 years, those with a diabetes duration of 6–10 years had a higher prevalence of poor glycaemic control (aPR = 1.13, 95% CI: 1.01–1.27; p = 0.037), and a significant linear trend was observed across the ordered duration categories in the unadjusted analysis (p for trend = 0.028). Compared with participants without any comorbidity, those with a single comorbidity (aPR = 1.20, 95% CI: 1.05–1.36; p = 0.007) and those with two or more comorbidities (aPR = 1.25, 95% CI: 1.09–1.43; p = 0.002) had a higher prevalence of poor glycaemic control. When the comorbidity components were analysed separately, hypertension was the only component independently associated with poor glycaemic control (aPR = 1.14, 95% CI: 1.02–1.28; p = 0.017), whereas reduced eGFR and overweight/obesity showed no significant association (Tables 3, 4, 5).

**TABLE 3:** Distribution of participant characteristics by glycaemic control status (*N* = 497).

Characteristic	Good glycaemic control, *n* (%)	Poor glycaemic control, *n* (%)
Overall	**104 (20.9)**	**393 (79.1)**
Gender		
Male	48 (17.6)	225 (82.4)
Female	56 (25.0)	168 (75.0)
Age		
Below 40	20 (33.9)	39 (66.1)
40–49	28 (21.9)	100 (78.1)
50–59	31 (19.3)	130 (80.7)
60 and above	25 (16.8)	124 (83.2)
Education		
Unable to read and write	30 (23.4)	98 (76.6)
Primary	27 (26.2)	76 (73.8)
Secondary	29 (21.0)	109 (79.0)
Higher secondary	9 (18.4)	40 (81.6)
Graduation and above	9 (11.4)	70 (88.6)
Residence		
Urban	46 (19.4)	191 (80.6)
Rural	58 (22.3)	202 (77.7)
Duration of diabetes (years)		
1–5	59 (26.0)	168 (74.0)
6–10	30 (16.4)	153 (83.6)
> 10	15 (17.2)	72 (82.8)
Family history of diabetes		
No	82 (22.1)	289 (77.9)
Yes	22 (17.5)	104 (82.5)
Comorbidity status		
No comorbidity	45 (32.8)	92 (67.2)
Single comorbidity	40 (17.5)	188 (82.5)
Two or more comorbidities	19 (14.4)	113 (85.6)
Complication status		
No complication	18 (19.1)	76 (80.9)
Single complication	63 (25.1)	188 (74.9)
Two or more complications	23 (15.1)	129 (84.9)
Sleep duration (hour)		
6–8	65 (22.8)	220 (77.2)
< 6	30 (17.5)	141 (82.5)
> 8	9 (22.0)	32 (78.0)
Smoking status		
Never	80 (21.3)	295 (78.7)
Current smoker	24 (19.7)	98 (80.3)
Physical exercise		
Regular	65 (20.5)	252 (79.5)
Irregular	39 (21.7)	141 (78.3)

*Note:* Values are presented as *n* (%). Glycaemic control was defined using HbA1c (good control < 7%; poor control ≥ 7%).

Abbreviation: T2DM = Type 2 diabetes mellitus.

**TABLE 4:** Crude and adjusted prevalence ratios for factors associated with poor glycaemic control (modified Poisson regression with robust variance).

Characteristic	Crude PR (95% CI)	Adjusted PR (95% CI)	*p*
Gender			
Male	1.00 (Ref.)	1.00 (Ref.)	
Female	0.91 (0.83–1.00)	0.94 (0.84–1.05)	0.281
Age (years)			
Below 40	1.00 (Ref.)	1.00 (Ref.)	
40–49	1.18 (0.96–1.45)	1.13 (0.93–1.38)	0.219
50–59	1.22 (1.00–1.49)	1.16 (0.94–1.41)	0.161
60 and above	1.26 (1.03–1.53)	1.14 (0.92–1.40)	0.231
Education			
Unable to read and write	1.00 (Ref.)	1.00 (Ref.)	
Primary	0.96 (0.83–1.12)	0.97 (0.84–1.12)	0.658
Secondary	1.03 (0.91–1.17)	1.02 (0.90–1.17)	0.715
Higher secondary	1.07 (0.91–1.26)	1.02 (0.87–1.20)	0.788
Graduation and above	1.16 (1.02–1.31)	1.13 (0.98–1.32)	0.094
Residence			
Urban	1.00 (Ref.)	1.00 (Ref.)	
Rural	0.96 (0.88–1.06)	0.96 (0.87–1.06)	0.413
Duration of diabetes (years)			
1–5	1.00 (Ref.)	1.00 (Ref.)	
6–10	1.13 (1.02–1.25)	**1.13 (1.01–1.27)**	**0.037**
> 10	1.12 (0.99–1.26)	1.11 (0.97–1.28)	0.133
Family history of diabetes			
No	1.00 (Ref.)	1.00 (Ref.)	
Yes	1.06 (0.96–1.17)	1.02 (0.92–1.13)	0.720
Comorbidity status			
No comorbidity	1.00 (Ref.)	1.00 (Ref.)	
Single comorbidity	1.23 (1.08–1.40)	**1.20 (1.05–1.36)**	**0.007**
Two or more comorbidities	1.27 (1.11–1.46)	**1.25 (1.09–1.43)**	**0.002**
Complication status			
No complication	1.00 (Ref.)	1.00 (Ref.)	
Single complication	0.93 (0.82–1.05)	0.90 (0.80–1.02)	0.113
Two or more complications	1.05 (0.93–1.18)	0.99 (0.86–1.15)	0.934
Sleep duration (hour)			
6–8	1.00 (Ref.)	1.00 (Ref.)	
< 6	1.07 (0.97–1.17)	1.05 (0.94–1.18)	0.380
> 8	1.01 (0.85–1.20)	1.02 (0.86–1.20)	0.856
Smoking status			
Never	1.00 (Ref.)	1.00 (Ref.)	
Current smoker	1.02 (0.92–1.13)	0.97 (0.87–1.09)	0.647
Physical exercise			
Regular	1.00 (Ref.)	1.00 (Ref.)	
Irregular	0.99 (0.90–1.08)	0.92 (0.83–1.02)	0.120

*Note:* Estimates were obtained from modified Poisson regression with robust (sandwich) variance, with poor glycaemic control (HbA1c ≥ 7%) as the outcome. Crude PRs are from single‐predictor models; adjusted PRs are mutually adjusted for all listed variables. Bold *p*‐values are < 0.05.

Abbreviations: CI, confidence interval; PR, prevalence ratio; Ref., reference category.

**TABLE 5:** Adjusted prevalence ratios for individual comorbidity components in relation to poor glycaemic control (modified Poisson regression with robust variance).

Comorbidity component	Adjusted PR (95% CI)	*p*	Adjusted PR (95% CI)^ᵃ^	*p*
Hypertension	**1.14 (1.02–1.28)**	**0.017**	**1.17 (1.05–1.30)**	**0.005**
Reduced eGFR	1.10 (0.96–1.26)	0.183	1.10 (0.96–1.26)	0.185
Overweight/obesity	1.09 (0.98–1.21)	0.115		

*Note:* Each model was adjusted for gender, age, education, residence, diabetes duration, family history, complication status, sleep duration, smoking, and physical exercise.

Abbreviations: CI, confidence interval; PR, prevalence ratio.

^ᵃ^ Sensitivity model excluding overweight/obesity. Bold values are *p* < 0.05.

### 3.3 Distribution of Complication Status by Glycaemic Control Status

**FIGURE 3:**
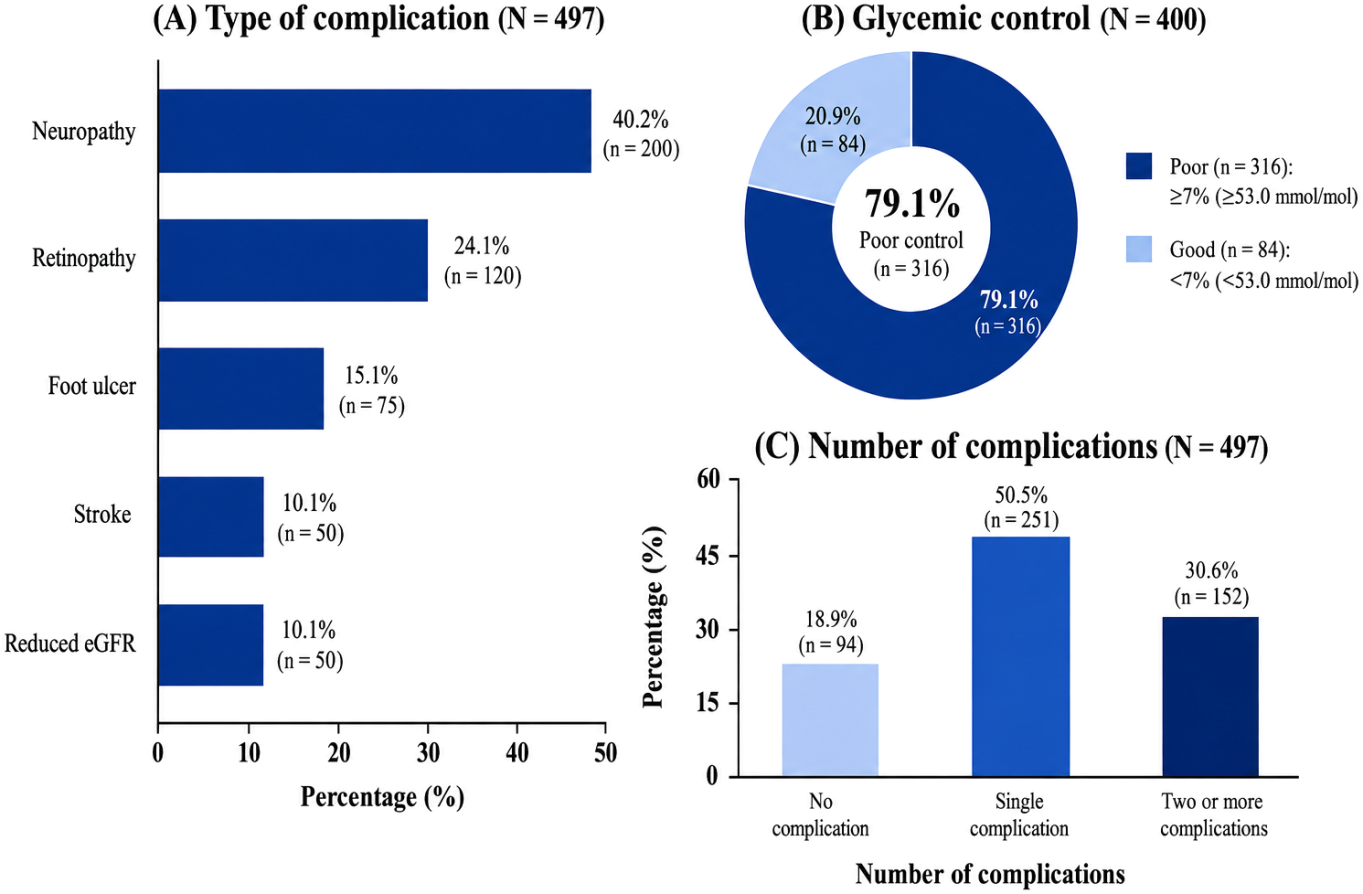
Glycaemic control (poor, HbA1c ≥ 7%; good, HbA1c < 7%) and diabetes‐related complications among T2DM patients (*N* = 497). (A) Complication types (not mutually exclusive); (B) glycaemic control status; (C) number of co‐occurring complications.

Poor glycaemic control was highly prevalent (79.1%), and most patients had at least one diabetes‐related complication, with neuropathy being the most common and 30.6% having multiple complications (Figure 3).

### 3.4 Distribution of Duration of T2DM by Glycaemic Control Status

**FIGURE 4:**
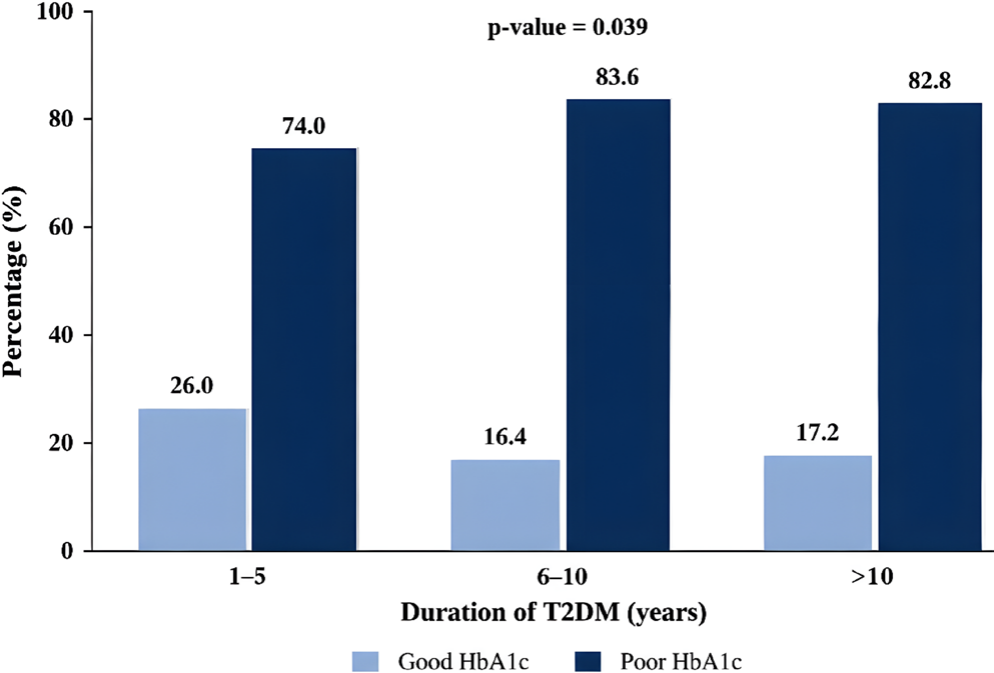
Distribution of duration of T2DM by glycaemic control status.

Poor glycaemic control was mostly observed among T2DM patients with the duration of disease between 6 to 10 years (83.6%), followed by more than 10 years (82.8%) and 1–5 years (74.0%), respectively. The association between duration of T2DM and the glycaemic control status was significant (*p*‐value = 0.039) (unadjusted Pearson *χ* ^2^ test; this figure presents an unadjusted comparison and should not be interpreted as confirming the adjusted association reported in Table 4); (Figure 4).

## 4 Discussion

This study aimed to assess the prevalence and factors associated with poor glycaemic control among patients with T2DM attending Jhenaidah Diabetic Hospital. The findings revealed that the vast majority of participants had HbA1c levels ≥ 7% and were classified as having poor glycemic control. Additionally, T2DM duration and the presence of comorbidities was identified as an independent factor significantly associated with poor glycemic control. The present study found that nearly four‐fifths (79.1%) of patients with T2DM had poor glycaemic control, a finding consistent with previous Bangladeshi studies that reported comparable rates of 81.2% [32], 87.6% [13], 71.8% [15], and 82.7% [16]. The high prevalence of poor glycaemic control may be attributed to factors such as limited access to consistent healthcare, insufficient medication adherence, and unsatisfactory lifestyle management among patients with T2DM in Bangladesh [15, 16].

However, a recent study conducted among T2DM outpatients in Dhaka, the capital city of Bangladesh [33], reported that only 33% of patients had poor glycaemic control, which is substantially lower than the prevalence observed in the present study. This disparity can be partially explained by differences in sociodemographic factors like monthly income and education level. The earlier research showed that a significant proportion of participants had higher degrees of education and moderate to good knowledge of diabetes [33], suggesting greater understanding and self‐management abilities; whereas only a small portion of participants in our study possessed a higher degree (9%). In addition, the higher percentage of patients with comparatively better household income in that study may have had greater access to healthcare, treatment compliance, and healthier lifestyle, which could have contributed to improved glycaemic control [33].

Recent studies from the South Asian region have also reported a high prevalence of poor glycaemic control, with rates of 76.6% in India [34], 66.4% in Nepal [35], and 83% in Pakistan [36] which supports the findings of the present study. However, the slight variation in prevalence across countries may be explained by differences in demographic characteristics, access to healthcare, and the quality of diabetes management services. Furthermore, heterogeneity in the methods used to assess glycaemic control may partly explain these discrepancies, as fasting blood glucose (FBG) and HbA1c reflect different aspects of glycaemic status and may produce different prevalence estimates [37]. Our study found that a longer duration of diabetes was significantly associated with poor glycaemic control. This outcome aligned with previous investigations indicating that patients with a prolonged duration of T2DM exhibited greater levels of HbA1c [38, 39]. A longer period of diabetes is frequently linked to poor glycaemic control, probably due to the progressive nature of the disease, a gradual deterioration of pancreatic β‐cell function, heightened insulin resistance, and diminished response to pharmacological interventions, often requiring escalated dosages and combination treatment strategies [40, 41].

Consistent with earlier studies, this study found that patients with one or more comorbidities had a significantly higher prevalence of poor glycaemic control as compared to those with no comorbidities [42]. Another study conducted among Colombian adults reported similar findings, showing that patients with T2DM with chronic kidney disease (CKD) had 78% higher odds of poor glycaemic control, while those with obesity had 52% higher odds [43]. The presence of multiple comorbidities (e.g., hypertension, cardiovascular disease, and obesity) increases treatment complexity [42], requiring concurrent management of multiple medications, dietary restrictions, and lifestyle modifications. This difficulty could negatively impact adherence to diabetes treatment and dietary guidelines, resulting in suboptimal glycaemic control [16]. In clinical practice, acute or severe comorbidities often get priority, thereby leading to unsatisfactory HbA1c management. Additionally, several comorbidities can directly impair insulin sensitivity and glucose metabolism [44], further complicating glycaemic control despite treatment. Multiple medications may also disrupt glucose control and elevate the risk of hypoglycaemia [45]. Moreover, comorbidities are often associated with fatigue, depression, and physical limitations ([46, 47]), which adversely affect diet, physical activity, and glucose monitoring, collectively contributing to poor glycaemic control. To our knowledge, this study provides the first evidence on the prevalence of and factors associated with poor glycaemic control among T2DM patients in the Jhenaidah district, a setting that is under‐represented in the Bangladeshi diabetes literature. Several limitations should be considered when interpreting these findings. First, participants were recruited using convenience sampling from a single referral hospital, so the results mainly apply to similar clinical settings and should be generalized to the wider population withT2DM with caution. Second, the cross‐sectional design can identify factors linked to poor glycaemic control but cannot establish cause and effect. In particular, because diabetes‐related complications may themselves be consequences of prolonged hyperglycaemia, adjusting for them in the same model may introduce over‐adjustment or collider bias, so the complication estimates should be interpreted cautiously. Because poor control was common, prevalence ratios were reported using modified Poisson regression, which is suitable for common outcomes. Third, some factors, such as medication type, insulin use, and treatment adherence, were not available, so residual confounding cannot be ruled out. Finally, some variables were based on self‐report or routine records. In particular, HbA1c values were obtained from routine laboratory reports, and it could not be confirmed whether all tests were performed in the same laboratory or with an NGSP/IFCC‐standardized assay; factors that can influence HbA1c interpretation, such as anaemia, haemoglobinopathies, recent transfusion, and advanced renal disease, could not be accounted for, individualized HbA1c targets were unavailable, and a threshold sensitivity analysis could not be performed. Self‐reported measures such as physical exercise are also subject to recall and social desirability bias and to likely exposure misclassification. Despite these limitations, the study used a fairly large sample and HbA1c‐based assessment, and it adds useful evidence from an under‐studied setting.

## 5 Conclusion

This study revealed a high prevalence of poor glycaemic control among patients with T2DM at Jhenaidah Diabetic Hospital, which was significantly associated with diabetes duration and comorbidity status. Poor control was linked to a diabetes duration of 6–10 years and to having one or more coexisting conditions. However, the association was not significant for patients with more than 10 years of diabetes; therefore, longer diabetes duration cannot be assumed to uniformly increase the risk of poor glycaemic control. Among individual conditions, hypertension was most strongly associated with poor control. These findings support closer clinical monitoring and integrated management of coexisting conditions, particularly hypertension and longer diabetes duration, and argue for strengthening structured follow‐up and comorbidity care within specialised diabetes services. Because diet, medication adherence, diabetes knowledge, and health awareness were not measured, broader lifestyle and awareness recommendations are offered only as contextual public health considerations rather than direct findings of this study. Future large‐scale, multicentre and longitudinal studies incorporating medication, adherence, and behavioural data are needed to establish temporal relationships and to guide targeted interventions.

## Author Contributions

**Md. Sakhawot Hossain:** conceptualization, writing – original draft, methodology, formal analysis, data curation, investigation. **Md. Shahadoth Hossain:** writing – original draft, data curation, resources. **Quazi Mumtarina Tabassum:** methodology, resources. **Md. Hasan Al Banna:** writing – review and editing. **Md. Moshiuzzaman Adnan:** data curation, writing – original draft, formal analysis. **Md. Amanullah:** writing – review and editing. **Md. Shovon Al‐Fuad:** validation. **Muhammad Tayyab Arshad:** writing – review and editing. **Rakibul Islam:** conceptualization, writing – original draft, data curation, formal analysis, methodology, investigation. **Fardib Mahbub:** writing – review and editing. **Tanvir Ahmad:** conceptualization, supervision, resources, writing – review and editing. **Tasniya Akter:** writing – review and editing.

## Funding

This research received no specific grant from any funding agency in the public, commercial, or not‐for‐profit sectors.

## Conflicts of Interest

The authors declare no conflicts of interest.

## Data Availability

The data that support the findings of this study are available from the corresponding author upon reasonable request.
